# Collagen-binding C-type natriuretic peptide enhances chondrogenesis and osteogenesis

**DOI:** 10.1172/jci.insight.198959

**Published:** 2025-12-23

**Authors:** Kenta Hirai, Kenta Sawamura, Ryusaku Esaki, Ryusuke Sawada, Yuka Okusha, Eriko Aoyama, Hiroki Saito, Kentaro Uchida, Takehiko Mima, Satoshi Kubota, Hirokazu Tsukahara, Shiro Imagama, Masaki Matsushita, Osamu Matsushita, Yasuyuki Hosono

**Affiliations:** 1 Department of Pediatrics, Okayama University Graduate School of Medicine, Dentistry, and Pharmaceutical Sciences, Okayama, Japan.; 2 Department of Orthopaedic Surgery, Nagoya University Graduate School of Medicine, Nagoya, Japan.; 3 Department of Pharmacology, Okayama University Graduate School of Medicine, Dentistry, and Pharmaceutical Sciences, Okayama, Japan.; 4 Advanced Research Center for Oral and Craniofacial Sciences, Okayama University Dental School, Okayama, Japan.; 5 Department of Orthopaedic Surgery, Kitasato University School of Medicine, Sagamihara, Japan.; 6 Department of Medical Technology, Faculty of Health Sciences, Ehime Prefectural University of Health Sciences, Ehime, Japan.; 7 Department of Biochemistry and Molecular Dentistry and; 8 Department of Bacteriology, Okayama University Graduate School of Medicine, Dentistry, and Pharmaceutical Sciences, Okayama, Japan.

**Keywords:** Bone biology, Cell biology, Development, Bone disease, Collagens, Orthopedics

## Abstract

C-type natriuretic peptide (CNP) is known to promote chondrocyte proliferation and bone formation; however, CNP’s extremely short half-life necessitates continuous intravascular administration to achieve bone-lengthening effects. Vosoritide, a CNP analog designed for resistance to neutral endopeptidase, allows for once-daily administration. Nonetheless, it distributes systemically rather than localizing to target tissues, which may result in adverse effects such as hypotension. To enhance local drug delivery and therapeutic efficacy, we developed a potentially novel synthetic protein by fusing a collagen-binding domain (CBD) to CNP, termed CBD-CNP. This fusion protein exhibited stability under heat conditions and retained the collagen-binding ability and bioactivity as CNP. CBD-CNP localized to articular cartilage in fetal murine tibiae and promoted bone elongation. Spatial transcriptomic analysis revealed that the upregulation of chondromodulin expression may contribute to its therapeutic effects. Treatment of CBD-CNP mixed with collagen powder to a fracture site of a mouse model increased bone mineral content and bone volume compared with CNP-22. Intraarticular injection of CBD-CNP to a mouse model of knee osteoarthritis suppressed subchondral bone thickening. By addressing the limitations of CNP’s rapid degeneration, CBD-CNP leverages its collagen-binding capacity to achieve targeted, sustained delivery in collagen-rich tissues, offering a promising strategy for enhancing chondrogenesis and osteogenesis.

## Abstract 2

Collagen-binding C-type natriuretic peptide localized to collagen-rich tissues and promoted bone elongation and fracture healing, and mitigated joint degradation.

## Introduction

C-type natriuretic peptide (CNP) is a peptide hormone secreted by the central nervous system, reproductive organs, and cardiovascular endothelium (1). It is initially synthesized as a 126-amino acid precursor protein (prepro-CNP), which is subsequently processed into 2 active forms: a 53-amino acid cyclic peptide (CNP-53) and a 22-amino acid peptide (CNP-22) (2, 3). CNP plays a multifaceted role in promoting osteoblast differentiation (4) and cartilage cell proliferation (5). CNP inhibits the downstream pathway of fibroblast growth factor receptor 3 (FGFR3) via natriuretic peptide receptor B (NPR-B) receptor (5) and is being applied for bone lengthening in achondroplasia. However, native CNP is rapidly cleared from the circulation by natriuretic peptide receptor C (NPR-C) and neutral endopeptidase (NEP), limiting its therapeutic potential (6). Notably, the half-life of CNP-22 in humans is only 2.6 minutes (7). Owing to this rapid degradation, bolus administration of CNP-22 renders it ineffective, and continuous intravascular infusion is required to elicit bone growth–promoting effects (8).

Vosoritide is a CNP analog modified with 2 additional N-terminal amino acids (Pro-Gly) on a 37-residue peptide backbone (9). It was designed to resist degradation by NEP and, when administered via once-daily s.c. injection, has been shown in clinical trials to enhance endochondral bone growth and ameliorate short stature in achondroplasia (10, 11). Currently, vosoritide is the only drug approved in the United States and Europe to improve growth in children with achondroplasia. However, vosoritide still demonstrates a relatively short half-life of 27.9 minutes in pediatric patients (12). Another long-acting CNP analogue developed to extend the bioactivity, TransCon CNP, is a prodrug consisting of CNP-38 (the 38 C-terminal amino acids of human CNP-53) conjugated to a polyethylene glycol (PEG) moiety via a cleavable linker, enabling sustained release of CNP-38 (13). In cynomolgus monkeys, the half-life of released CNP-38 was approximately 90 hours (13). It has shown promise in clinical trials as a once-weekly treatment option for children with achondroplasia (14, 15) and is currently in phase 2 clinical trial. Nevertheless, native CNP and long-acting CNP analogs are limited by widespread distribution, which prevent them from remaining localized in target tissues.

The collagen-binding domain (CBD) is a structural component of collagenase derived from *Hathewaya histolytica* (formerly *Clostridium histolyticum*), a pathogen associated with gas gangrene (16–18). We have previously developed a drug delivery system using CBD fused with fibroblast growth factor 2 (FGF2) to enhance its retention and efficacy in collagen-rich tissues (19–21), using CBD as an anchoring module to localize therapeutic activity (22). Building on this strategy, we have synthesized a potentially novel fusion protein comprising CNP linked to CBD (CBD-CNP), designed to bind to collagen matrix, improve local stability, and sustain efficacy within the collagen scaffold. This study aimed to evaluate the chondrogenic and osteogenic effects of CBD-CNP and to assess its potential as a drug delivery system targeted for collagen-rich tissues.

## Results

### Purification and 3-dimensional structural prediction of CBD-CNP.

**Figure 1:**
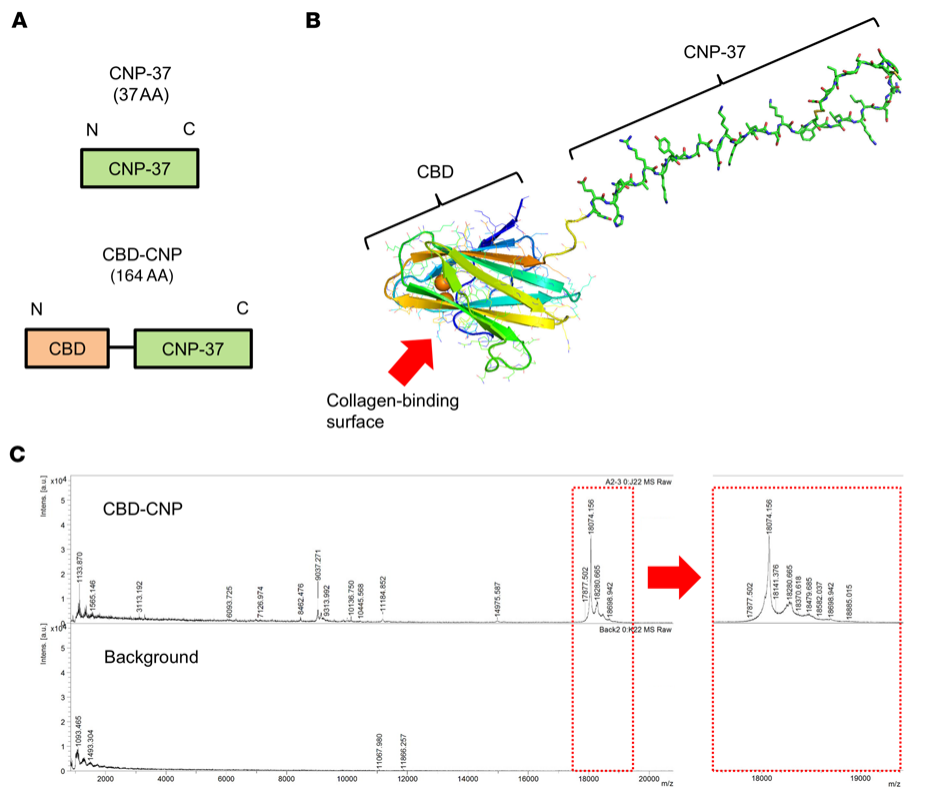
Design, 3-dimensional structural prediction, and molecular weight of CBD-CNP. (**A**) Design of CBD-CNP. The collagen-binding domain (CBD) is placed at the N-terminus and CNP-37 at the C-terminus, connected by a Gly-Gly-Ser-Pro-Gly spacer. (**B**) Three-dimensional structural prediction of CBD-CNP using AlphaFold3. (**C**) Molecular mass of CBD-CNP measured by MALDI-TOF-MS. CBD, collagen-binding domain; CNP, C-type natriuretic peptide; AA, amino acids; MALDI-TOF-MS, matrix-assisted laser desorption/ionization time-of-flight mass spectrometry.

CBD-CNP was designed as illustrated in Figure 1A, with a CBD placed at the N-terminus and CNP-37 at the C-terminus, connected via a flexible Gly-Gly-Ser-Pro-Gly spacer. The full amino acid sequence is shown in Supplemental Figure 1 (supplemental material available online with this article; https://doi.org/10.1172/jci.insight.198959DS1). Structural prediction using AlphaFold3 revealed a β-sandwich structure corresponding to the CBD at the N-terminus of the fusion protein (Figure 1B). Two calcium ions (shown as orange spheres) within the CBD (Protein Data Bank ID 1NQD) were accurately predicted. The collagen-binding surface was localized to the lower left, whereas the spacer (yellow) and CNP moiety (green) connected to a β sheet separate from the collagen-binding site. Therefore, there would be no interference between the bound collagen and CNP. A disulfide bond (yellow stick) of Cys residues was observed at the C-terminus of CNP. Matrix-assisted laser desorption/ionization time-of-flight mass spectrometry (MALDI-TOF-MS) confirmed the molecular mass of CBD-CNP as 18,074 Da, consistent with the theoretical value (Figure 1C).

### CBD-CNP retained collagen-binding capacity even after heat processing.

**Figure 2:**
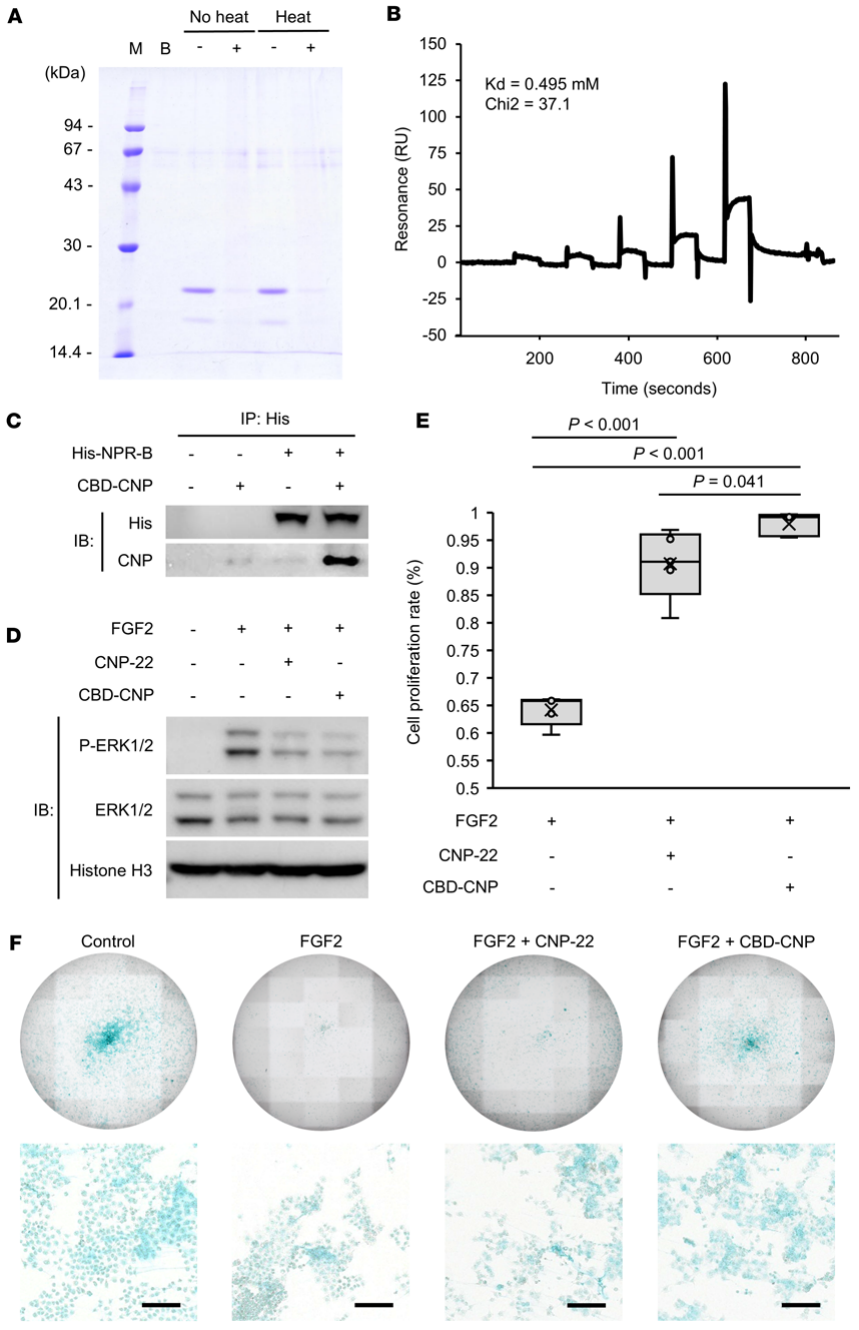
Collagen and ligand-receptor binding ability, and chondrocyte proliferation of CBD-CNP. (**A**) Collagen-binding ability and thermal stability of CBD-CNP. Unbound CBD-CNP was assessed by SDS-PAGE. M, molecular weight marker; B, buffer control; No heat, no preheating; Heat, preheating at 56°C 30 minutes; (–), no collagen powder was added; (+), collagen powder was added. (**B**) Surface plasmon resonance analysis of CBD-CNP binding to recombinant NPR-B immobilized on a CM5 chip. The Kd value was calculated from sensorgrams as described under the experimental procedures. (**C**) Ligand-receptor binding assay with CBD-CNP and NPR-B, the receptor for CNP. (**D**) Inhibitory effect of FGF2-FGFR3 downstream signaling pathway of RCS cells owing to the binding of CNP to NPR-B. Phosphorylation of ERK1/2 in RCS cells was assessed by FGF2 alone and combined treatment with CNP-22 or CBD-CNP. (**E**) Cell proliferation assessment of RCS cells after treatment with FGF2 alone or in combination with CNP-22 or CBD-CNP (*n* = 5). The cell proliferation rate was calculated based on the cell count with no drug treatment as 100%. (**F**) Alcian blue staining of RCS cells in each group. Scale bars: 200 μm in **F**. Statistical analysis was performed by using 1-way ANOVA followed by Tukey post hoc tests. SDS-PAGE, sodium dodecyl sulfate polyacrylamide electrophoresis; RU, response unit; IP, immunoprecipitation; IB, immunoblot; NPR-B, natriuretic peptide receptor B; CNP, C-type natriuretic peptide; FGF2, fibroblast growth factor 2; FGFR3, fibroblast growth factor receptor 3; RCS, rat chondrosarcoma; ERK, extracellular signal-regulated kinase; P-ERK, phospho-extracellular signal-regulated kinase.

To evaluate collagen-binding ability and thermal stability, CBD-CNP was incubated with collagen powder. Unbound CBD-CNP was analyzed by sodium dodecyl sulfate polyacrylamide electrophoresis (SDS-PAGE). When CBD-CNP was mixed with collagen powder, it was retained on the filter, and no band was detected with the solvent that passed through the filter by SDS-PAGE, indicating that CBD-CNP bound to collagen (Figure 2A). This result was consistent regardless of whether CBD-CNP was preheated at 56°C for 30 minutes, suggesting that the protein retained its structural integrity and collagen-binding capacity even after thermal treatment. These findings demonstrate that CBD-CNP possesses strong collagen-binding capability and exhibits stability under heat conditions.

### CBD-CNP binds to the CNP receptor and induces downstream signaling.

Surface plasmon resonance analysis confirmed the interaction between CBD-CNP and NPR-B, the receptor for CNP. Recombinant NPR-B was immobilized on a CM5 chip, and binding with CBD-CNP was assessed (Figure 2B). The analysis yielded a dissociation constant of 0.495 mM, indicating a measurable affinity between CBD-CNP and NPR-B. To further validate this interaction, an in vitro protein binding assay was performed using His-tagged NPR-B and CBD-CNP. CBD-CNP was detected only in samples containing both proteins, supporting its specific binding to NPR-B (Figure 2C). To assess the functional consequence of this interaction, we examined the effect of FGF2-mediated FGFR3 signaling, which is known to be inhibited by CNP via NPR-B. In rat chondrosarcoma (RCS) cells, phosphorylation of extracellular signal-regulated kinase (ERK) 1/2 induced by FGF2 was suppressed upon cotreatment with either CNP-22 or CBD-CNP (Figure 2D). These findings indicate that the CBD moiety does not impair the activity of CNP.

### CBD-CNP enhances proliferation of RCS cells.

RCS cell proliferation rate was measured following treatment with FGF2, CNP-22, or CBD-CNP, with the untreated control set as 100% for comparison. FGF2 reduced cell proliferation compared with control (64.2% ± 2.7%), whereas cotreatment with CNP-22 (*P* < 0.001) or CBD-CNP (*P* < 0.001) significantly improved cell proliferation rate, with CBD-CNP showing a more pronounced effect (90.7% ± 6.3% for CNP-22 versus 98.0% ± 2.1% for CBD-CNP, *P* = 0.041; Figure 2E). Alcian blue staining of RCS cells demonstrated abundant cartilage-related proteoglycan, and FGF2 suppressed proteoglycan production and induced extracellular matrix degradation (23), resulting in weak staining. In contrast, cotreatment with CBD-CNP restored the Alcian blue staining (Figure 2F).

### CBD-CNP promotes sustained bone growth in fetal murine tibiae treated with FGF2.

**Figure 3:**
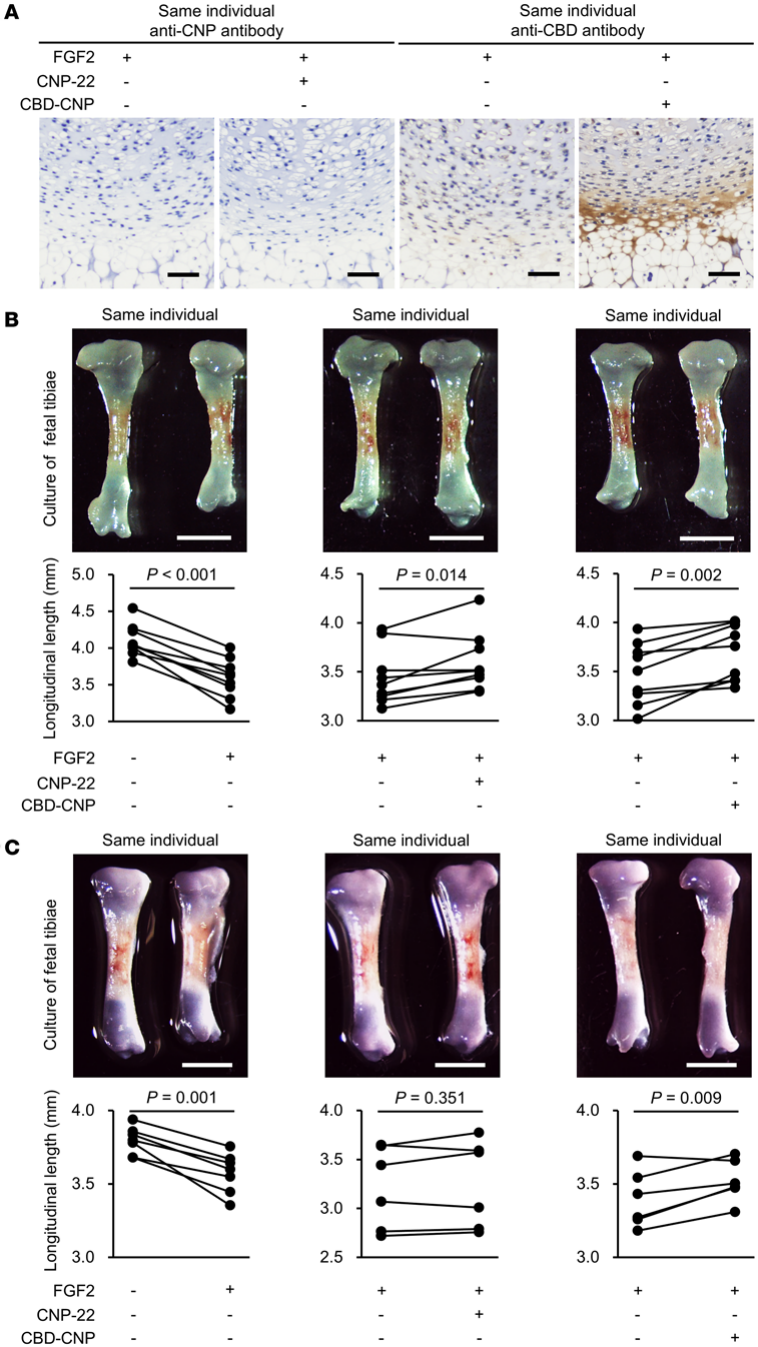
Bone growth effect of CBD-CNP in ex vivo bone culture. (**A**) Representative DAB staining images of fetal murine tibiae (E16.5, FVB background) treated with FGF2 in the presence of CNP-22 or CBD-CNP with daily medium changes. CNP-22 was detected with anti-CNP antibody, and CBD-CNP was detected with anti-CBD antibody. (**B**) Fetal murine tibiae were cultured and treated with FGF2 in the presence of CNP-22 or CBD-CNP with daily medium changes. After 4 days, the tibial lengths were measured and compared with those of contralateral untreated tibiae in the same patients (*n* = 9). (**C**) To assess the prolonged effects, only a single administration of CNP-22 or CBD-CNP was performed on the first day, whereas FGF2 was added daily. The tibial lengths were measured after 4 days from the first dosage (*n* = 7 in FGF2 and CBD-CNP groups, and *n* = 6 in CNP-22 group). Scale bars: 50 μm in **A**; 1 mm in **B** and **C**. The tibial lengths within patients were compared using a paired *t* test. DAB, 3,3′-diaminobenzidine.

The bone elongation effect of CBD-CNP was evaluated in ex vivo fetal murine (embryonic day 16.5, FVB background) tibiae cultured with FGF2, CNP-22, or CBD-CNP. Histological analysis using 3,3’-diaminobenzidine (DAB) staining demonstrated cartilage-specific staining with anti-CBD antibody in CBD-CNP–treated tibiae but no staining with anti-CNP antibody in CNP-22-treated tibiae (Figure 3A). FGF2 significantly inhibited bone growth compared with untreated contralateral tibiae of the same patients (4.10 ± 0.22 mm versus 3.59 ± 0.26 mm, *P* < 0.001; Figure 3A). Daily administration of CNP-22 (3.45 ± 0.29 mm versus 3.59 ± 0.30 mm, *P* = 0.014) or CBD-CNP (3.48 ± 0.31 mm versus 3.70 ± 0.29 mm, *P* = 0.002) significantly increased the length of FGF2-treated tibiae (Figure 3B). Although a single dose of CNP-22 did not significantly enhance tibial length (3.21 ± 0.42 mm versus 3.25 ± 0.45 mm, *P* = 0.351), a single administration of CBD-CNP led to a sustained bone elongation effect (3.49 ± 0.30 mm versus 3.61 ± 0.27 mm, *P* = 0.009; Figure 3C).

### Spatial transcriptomics revealed the pharmacological effects of CBD-CNP against FGF2.

**Figure 4:**
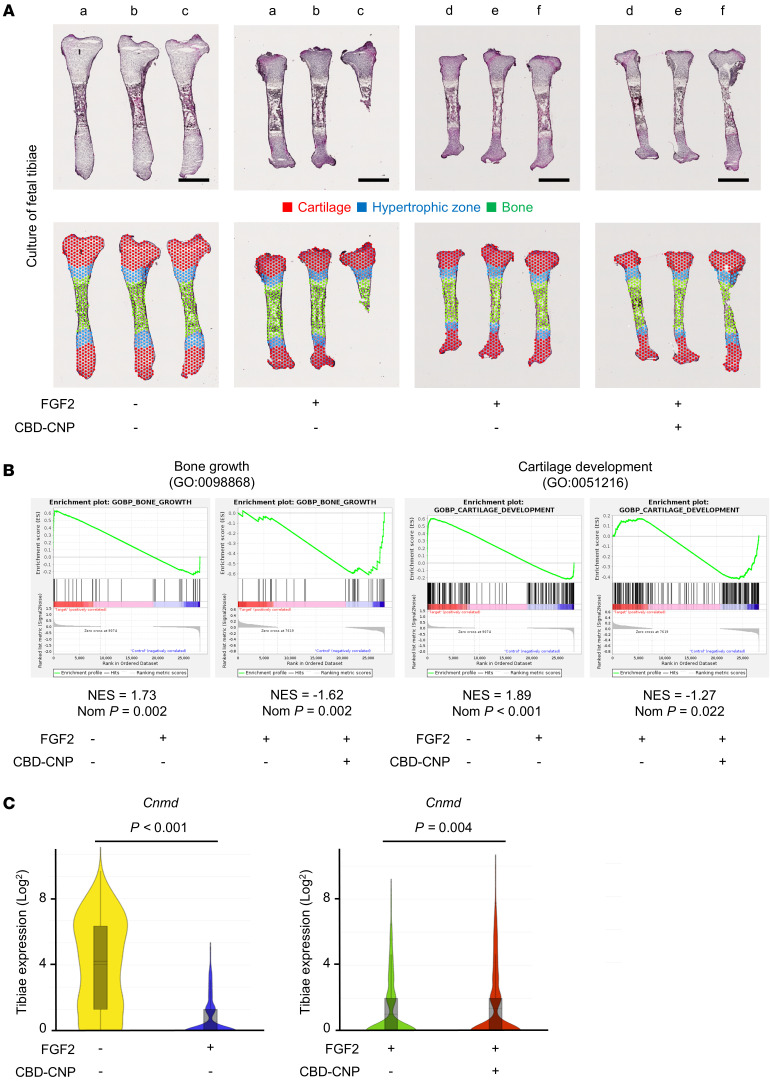
Pharmacological effects of CBD-CNP on bone growth revealed with spatial transcriptomics. (**A**) H&E staining and sectioning of Visium Spatial Gene Expression Slides of fetal murine tibiae. The tibiae labeled by letter indicates samples from the same patient. (**B**) Gene set enrichment analysis of hypertrophic zones in spatial transcriptomics. (**C**) Violin plots of gene expression in total tibiae. Scale bars: 1 mm in **A**. Statistical analysis was performed using the Student’s *t* test. NES, normalized enrichment score; Nom, nominal; *Cnmd*, chondromodulin.

Spatial transcriptomic analysis of fetal tibiae was performed to elucidate the effect of CBD-CNP on FGF2. Visium transcriptomics–run data are summarized in Supplemental Table 1. H&E-stained images of fetal tibiae are shown in Figure 4A, with tibiae labeled by letter to indicate samples from the same patient. The tibiae were segmented into cartilage and bone regions, and the hypertrophic zone of cartilage was delineated in blue based on histology and gene expression patterns (Figure 4A) (24). Gene set enrichment analysis of the hypertrophic zone revealed that FGF2 administration downregulated pathways related to bone growth and cartilage development, whereas coadministration of CBD-CNP restored the expression of these pathways (Figure 4B). Additional pathways involved in chondrocyte development, differentiation, and growth plate cartilage formation during endochondral bone morphogenesis were also upregulated in the CBD-CNP–treated group (Supplemental Figure 2). Among gene expression profiles of total tibiae, chondromodulin (*Cnmd*), a cartilage-specific glycoprotein known to promote chondrocyte growth (25), was significantly downregulated by FGF2 (*P* < 0.001) but significantly upregulated by CBD-CNP coadministration (*P* = 0.004; Figure 4C). The increase in *Cnmd* expression is likely to contribute to the bone elongation effect observed with CBD-CNP treatment. Another bone formation marker, osteocalcin (*Bglap*), was also upregulated by CBD-CNP coadministration (*P* = 0.003). FGF2 administration significantly downregulated *Col1a* (*P* < 0.001), *Col2a* (*P* < 0.001), *Col10a* (*P* < 0.001), and *Alpl* (*P* < 0.001), but these genes were not upregulated by coadministration of CBD-CNP. All gene expression profiles are provided in the supporting data value XLSX file.

### CBD-CNP promotes bone healing in a murine fracture model.

**Figure 5:**
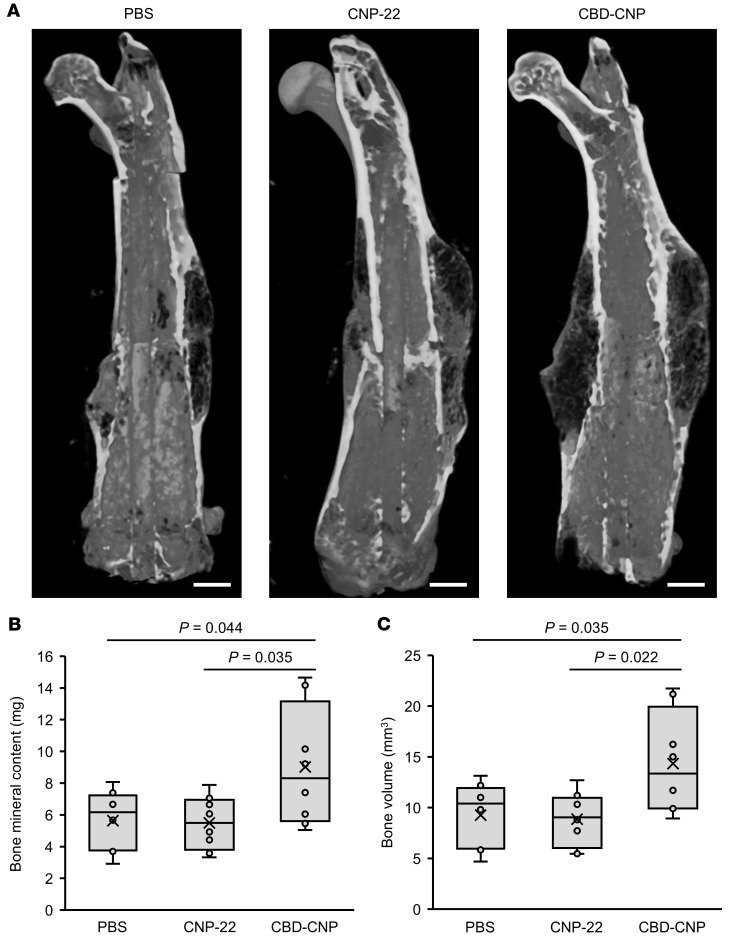
CBD-CNP increases bone volume in the C57BL/6J mouse model of bone fracture. (**A**) Representative μCT images of the fracture site of femurs treated with collagen powder mixed with either PBS, CNP-22, or CBD-CNP. (**B** and **C**) Bone mineral content and bone volume of each group measured by μCT images (*n* = 8). Scale bars: 1 mm in **A**. Statistical analysis was performed by using 1-way ANOVA followed by Tukey post hoc tests. CT, computed tomography; PBS, phosphate-buffered saline.

As *Fgfr3*-deficient (–/–) mice exhibit accelerated fracture repair (26), we assessed bone regeneration of CBD-CNP using a murine fracture model. Collagen powder loaded with either phosphate-buffered saline (PBS), CNP-22, or CBD-CNP was applied to the fracture site of C57BL/6J mice. After 4 weeks, μCT analysis showed enhanced callus formation in the CBD-CNP group (Figure 5A). CBD-CNP significantly increased bone mineral content (BMC) (9.02 ± 3.77 mg) compared with CNP-22 (5.49 ± 1.68 mg, *P* = 0.035; Figure 5B). Similarly, bone volume (BV) was significantly higher in mice treated with CBD-CNP (14.34 ± 5.08 mm^3^) compared with those treated with CNP-22 (8.87 ± 2.59 mm^3^, *P* = 0.022; Figure 5C).

### Intraarticular CBD-CNP administration attenuates joint degradation.

**Figure 6:**
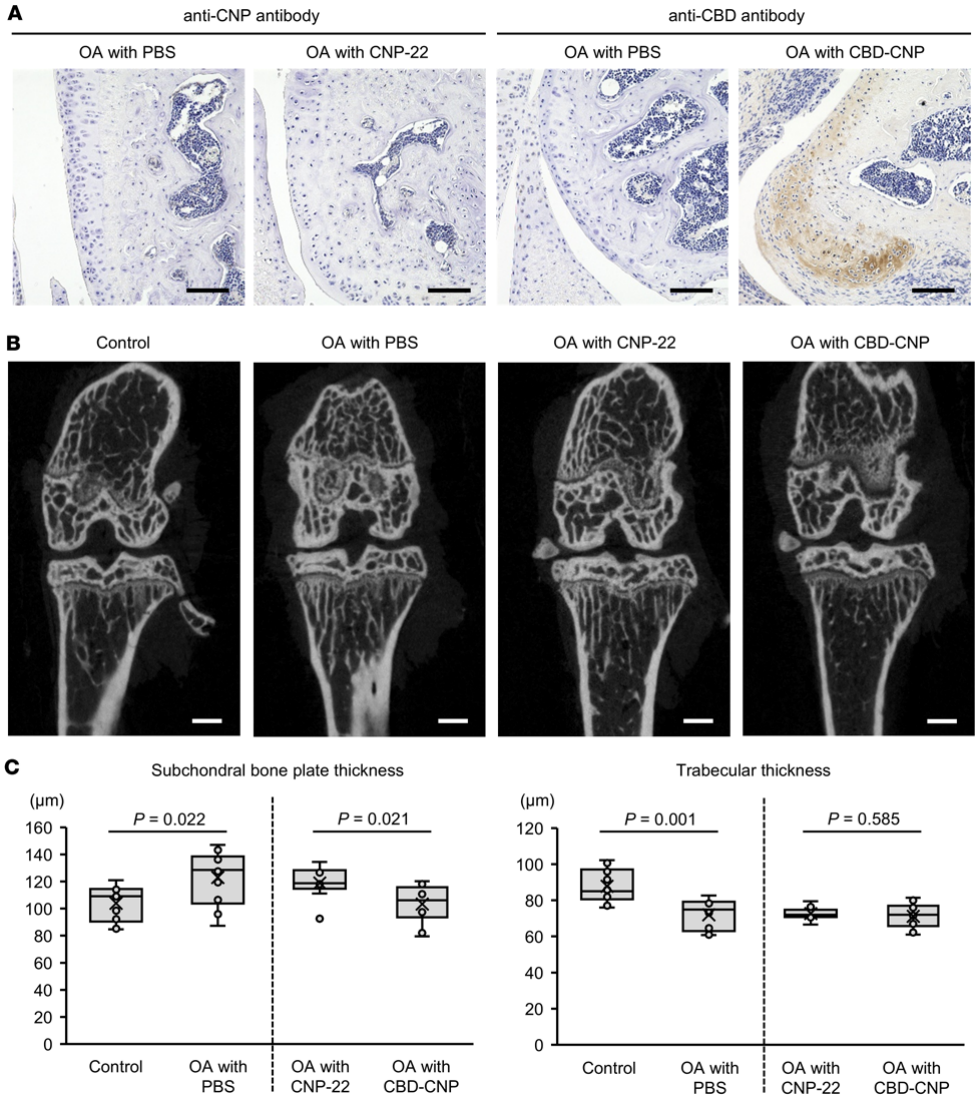
Intraarticular injection of CBD-CNP in the FVB/NJcl mouse model of knee osteoarthritis. (**A**) Representative DAB staining images of knee joint tissues from the mouse model of knee OA following intraarticular injection of PBS, CNP-22, or CBD-CNP. CNP-22 was detected with anti-CNP antibody, and CBD-CNP was detected with anti-CBD antibody. (**B**) Representative μCT images of the mouse model of knee OA with intraarticular injection of PBS, CNP-22, or CBD-CNP. The images of control and OA with PBS were obtained from the same patient. (**C**) Quantitative analyses of subchondral bone plate thickness and trabecular thickness using μCT images (*n* = 10 in control/OA with PBS, and OA with CBD-CNP groups, and *n* = 9 in OA with CNP-22 group). The data of control and OA with PBS were obtained from the same patients. Scale bars: 100 μm in **A**; 500 μm in **B**. Statistical analysis was performed using the Student’s *t* test. OA, osteoarthritis.

The persistence of CBD-CNP in joint tissues and its effects on bone morphology were evaluated using the FVB/NJcl mouse model of knee osteoarthritis (OA). DAB staining performed 3 days after the final intraarticular injection revealed no detectable signal with anti-CNP antibody in the articular cartilage of mice treated with CNP-22. In contrast, CBD-CNP–treated joints showed clear DAB staining with anti-CBD antibody in the articular cartilage even after 3 days of the last administration (Figure 6A). μCT analysis revealed that the subchondral bone plates in OA model mice were significantly thicker compared with the contralateral, nonoperated control side (*P* = 0.022). Intraarticular administration of CBD-CNP significantly improved this thickness compared with PBS (*P* = 0.023) and CNP-22 (*P* = 0.021; Figure 6, B and C). Although trabecular thickness was significantly reduced in the OA model mice (*P* = 0.001), there was no improvement observed after administration of CNP-22 or CBD-CNP. No significant differences were observed in other trabecular parameters, bone mineral density (BMD), and BV/total volume (BV/TV), between the OA model and the nonoperated control sides (Supplemental Figure 3).

## Discussion

In this study, we successfully purified a novel synthetic fusion protein, CBD-CNP, by linking CBD with CNP. Our results demonstrate that CBD-CNP retains the collagen-binding ability of CBD and the bioactivity of CNP, including its ability to promote chondrogenesis and osteogenesis. Importantly, CBD-CNP exhibits heat stability and prolonged retention within collagen-rich tissues following local administration, thereby overcoming the major limitation of endogenous CNP, its rapid inactivation, and short half-life. Moreover, CBD-CNP can be locally administered for site-specific delivery in collagen-containing tissues.

Vosoritide, a modified CNP analog, has entered clinical use for treating short stature in achondroplasia, but its systemic administration may lead to adverse effects such as hypotension (10, 11). In contrast, CBD-CNP is designed for localized application, which may reduce systemic adverse effects and enable targeted treatment, for instance, to 1 limb in congenital leg length discrepancy. In future studies, we would like to assess localized bone elongation effects via intraarticular administration of CBD-CNP in young mouse models. TransCon CNP, another long-acting CNP analogue, is a prodrug in which CNP-38 is conjugated to a PEG moiety via a cleavable linker. The use of large molecular weight PEG has been associated with cytoplasmic vacuolization in various tissues, raising safety concerns, particularly in pediatric patients (27).

Although systemic administration of bone morphogenetic protein 2 (BMP-2) has shown efficacy in promoting bone formation, it carries risks such as heterotopic ossification and carcinogenicity (28). In contrast, CBD-CNP offers a targeted and potentially safer alternative through local administration with collagen powder at fracture or osteotomy sites during osteosynthesis. CBD-CNP could promote early bone union through prolonged site-specific activation. This localized delivery approach may enhance therapeutic efficacy while minimizing systemic exposure, thereby reducing dosing frequency, total drug dose, treatment costs, and risks of adverse effects.

Spatial transcriptomic analysis of fetal tibiae demonstrated that CBD-CNP enhanced bone growth and cartilage development in the hypertrophic zone under FGF2 treatment (Figure 4B). Notably, among the differentially expressed genes, CBD-CNP significantly upregulated the expression of *Cnmd* (Figure 4C), a cartilage-specific glycoprotein known to stimulate proteoglycan synthesis in growth plate chondrocytes (29). A previous study reported that *Cnmd*-deficient (–/–) mice exhibit delayed fracture healing owing to impaired periosteal chondrocyte differentiation and reduced formation of endochondral osteoblasts (30). Moreover, loss of *Cnmd* function exacerbated OA progression by disrupting its antiangiogenic activity, leading to vascular invasion of the articular cartilage. Conversely, inducible *Cnmd* expression alleviated OA progression by inhibiting HIF-2α nuclear translocation and transcriptional activity (31). These findings suggest that the therapeutic benefits of CBD-CNP may be mediated, at least in part, by pharmacologically enhancing *Cnmd* expression, thereby supporting bone growth, fracture repair, and OA attenuation. Furthermore, given that *Cnmd* is necessary for traction of cartilage callus during endochondral bone formation (25), CBD-CNP–induced upregulation of *Cnmd* may also be beneficial for promoting bone union in distraction osteogenesis for achondroplasia.

This study demonstrated that intraarticular administration of CBD-CNP improved the thickness of subchondral bone plates in knee OA. These findings are supported by a previous report showing that transgenic overexpression of CNP in chondrocytes can protect against cartilage damage in a mouse model of inflammatory arthritis (32). Current treatment options for OA, a representative disease of articular cartilage disorders, are largely limited to symptom management. These include oral nonsteroidal antiinflammatory drugs (NSAIDs) for analgesia, intraarticular injections of hyaluronic acid or steroids, and ultimately, artificial joint replacement surgery (33). Although oral NSAIDs are effective for pain relief, long-term use is associated with gastrointestinal disorders. Intraarticular corticosteroid injections offer temporary analgesia, but long-term use may lead to cartilage degradation; notably, a 2-year administration of intraarticular triamcinolone resulted in cartilage volume loss without significant improvement in knee pain compared with saline (34). Although hyaluronic acid injections (viscosupplementation) are commonly employed for symptomatic OA with insufficient evidence, meta-analyses suggest only moderate pain reductions alongside increased risk of adverse events such as injection-site reactions and joint swelling (35). Mesenchymal stem cell injections may provide symptomatic relief but have not demonstrated consistent structural regeneration of articular cartilage (36). Artificial joint replacement surgery remains the definitive treatment for end-stage OA, yet it carries complications including bacterial infection, thromboembolism, and substantial socioeconomic burden. Importantly, no current therapy effectively halts or reverses structural joint degeneration. In this context, CBD-CNP represents a promising disease-modifying OA therapy that could bridge the treatment gap between symptomatic relief and surgical intervention, CBD-CNP may delay or obviate the need for artificial joint replacement in early to moderate stages of OA.

Compared with other collagen-binding proteins, CBD-CNP has a smaller molecular weight than FGF2-CBD (19–21), potentially offering advantages in terms of improved production efficiency and reduced antigenicity. Moreover, although previous CBD-fusion proteins typically positioned the bioactive domain at the N-terminus, CBD-CNP was uniquely designed with the bioactive molecule, CNP, at the C-terminal. This orientation preserves the collagen-binding functionality of the CBD moiety while maintaining the bioactivity of the C-terminal side of CNP. This novel fusion configuration represents a technical innovation, for bioactive substances whose functional domains reside at the C-terminus, by enabling collagen-targeting without compromising biological efficacy.

This study has some limitations to acknowledge. First, we were unable to compare CBD-CNP with vosoritide, owing to the unavailability of vosoritide for research use at the time this study began. Consequently, CNP-22 was selected as the comparator. Second, we were unable to evaluate the bone growth–promoting effects of CBD-CNP in vivo, as the joint cavity of juvenile *Fgfr3*^ach^ achondroplasia mouse models (37–39) was too small to allow intraarticular injection. Beyond *Fgfr3* overactivation, other genetic mechanisms such as CNP deficiency (40) and overexpression of FGF18 in the growth plate (41, 42) also cause dwarfism. Therefore, future studies using medium- to large-sized animal models of dwarfism that allow for intraarticular administration are warranted. As another strategy, we would also like to consider administering CBD-CNP locally after bone-lengthening surgery for achondroplasia or congenital leg length discrepancy, thereby promoting bone union and reducing pain and rehabilitation period associated with surgery. Third, because CBD is derived from a bacterial protein, concerns remain about the potential immunogenicity of CBD-CNP. Although repeated intraarticular injections were effective and no serious adverse effects were observed in this study, the production of anti–CBD-CNP antibodies in mice was not be evaluated. Furthermore, anti-drug antibody analysis in animals does not correlate with efficacy or safety in humans. As a method for evaluating human immunogenicity, we plan to use Interactive Screening and Protein Reengineering Interface (ISPRI) to predict T cell epitopes of protein therapeutics in silico and, if necessary, optimize high-risk amino acid sequences to reduce immunogenicity (43). Fourth, we used only male mice in this study, owing to established sex-specific differences in disease progression. OA progression and bone formation are influenced by female hormones, and ovariectomy in female mice inhibited bone formation (44). Further detailed toxicological assessments, optimization of local delivery strategies, and large-scale efficacy validation including female patients will be necessary before clinical application.

In conclusion, CBD-CNP addresses a key limitation of native CNP, its rapid inactivation, by enabling sustained, localized delivery through collagen-binding affinity in collagen-rich tissues. CBD-CNP is expected to hold a variety of clinical applications, including bone growth promotion, enhancement of fracture healing, and inhibition of joint degeneration.

## Methods

### Sex as a biological variable.

This study employed only male mice because OA progression and bone formation are influenced by female hormones (44), and further validation is required to extrapolate the findings of this study to female patients.

### Design of CBD-CNP.

To integrate CBD into a fusion protein, it was necessary to determine whether to fuse it to the N-terminus or C-terminus of the target moiety. Because the disulfide bridge at the C-terminus of CNP is essential for its biological activity, we decided to fuse CBD to the N-terminus. We also evaluated several candidate CBDs to identify the most suitable for fusion with CNP. Our previous structural studies identified the CBD derived from *H*. *histolytica* class I collagenase (ColG) as a β-sandwich (Protein Data Bank ID 1NQD) (18). The C-terminal residue Lys1008 of this CBD (s3b, ColG) is located on a β-sheet opposite the collagen-binding surface, with its carboxyl group exposed to the surrounding environment (18). Based on these characteristics, we selected the s3b domain of ColG for N-terminal fusion with CNP. To ensure that the fusion did not interfere with the function of either domain, we included a short spacer between CBD and CNP. CNP-22, comprising the 22 C-terminal residues, retains full biological activity, suggesting that 15 N-terminal residues of CNP-37 are nonessential and may function as a natural spacer. To further optimize expression efficiency and maintain structural flexibility, we inserted a 5-amino-acid spacer designed to provide independent movement and reduce steric hindrance. We placed 2 small glycine in the proximity of the CBD, a serine to provide hydration, a proline to provide mobility at a position slightly away from the CBD, and a small glycine residue. Based on the consideration of these factors, the spacer sequence, Gly-Gly-Ser-Pro-Gly, was chosen. The 3-dimensional structure of the CBD-CNP fusion protein was predicted using AlphaFold3 (45).

### Constructing expression plasmid of CBD-CNP fusion protein.

The DNA fragment encoding the CBD-CNP fusion protein was obtained by PCR amplification of the CBD (s3b, ColG) gene from the pCHG115 plasmid (18) as a template. The forward primer encoded the N-terminus of CBD, and the reverse primer encoded the C-terminus of CBD, the spacer, and CNP-37. This DNA fragment was inserted into the TA cloning vector pMD19, and sequencing confirmed the correct construction of the DNA fragment encoded to the fusion protein. The plasmid was then digested with restriction enzymes of *EcoRI* and *XhoI* to extract the target DNA fragment. The fragment was inserted into the same restriction enzyme sites in the pGEX-4T-2, which is the glutathione S-transferase (GST) fusion protein expression vector. This expression plasmid was designated pEPU005.

### Purification of CBD-CNP.

*E*. *coli* BL21 CodonPlus RIL (Agilent, Santa Clara, CA) cells were transformed with CBD-CNP fusion protein expression plasmid (pEPU005) and cultured in 4,000 mL of 2YT-G medium supplemented with 50 mg/mL ampicillin and 30 mg/mL chloramphenicol at 37°C with shaking at 150 rpm. Protein expression was induced with 1 mM isopropyl-β-D-thiogalactopyranoside. Cells were lysed twice using a French press at 10,000 psi. The lysate, free of cell debris, was mixed with glutathione-Sepharose beads (Cytiva) to bind the GST-fusion protein and then eluted with a solution containing glutathione. GST was cleaved with thrombin protease (Cytiva), and the mixture was dialyzed 3 times at 4°C against 1,000 mL of 50 mM Tris-HCl (pH 7.5), and 1 mM CaCl_2_. The solution was then mixed with glutathione-Sepharose beads, and CBD-CNP was eluted with 50 mM Tris HCl (pH 7.5), 200 mM NaCl, and 1 mM CaCl_2_. Mass spectrometric analysis of the purified CBD-CNP was performed using MALDI-TOF-MS using ultrafleXtreme (Bruker Daltonics).

### Collagen-binding assay.

To assess collagen-binding ability and thermal stability of CBD-CNP, 10 mg of collagen powder (Sigma, type I C-9879) was mixed with 100 μL solution containing 200 pmol of CBD-CNP, either untreated or preheated at 56°C for 30 minutes. The mixtures were placed in Ultrafree MC GV 0.22 μm filter cup (Millipore) and incubated at room temperature for 30 minutes. The filtrate was collected by centrifugation at 15,000 rpm for 5 minutes and unbound CBD-CNP was analyzed by SDS-PAGE.

### Surface plasmon resonance measurements.

Recombinant NPR-B (DIMA biotech) was diluted to 10 μg/mL in 10 mM sodium acetate buffer (pH 4.5) and immobilized onto CM5 sensor chips (Cytiva) using standard amine coupling protocols. CBD-CNP was diluted in buffer (50 mM Tris HCl, pH 7.5, 200 mM NaCl, 1 mM CaCl_2_) to concentrations of 1.25, 2.5, 5.0, 10, and 20 μM and injected into the flow cells. For affinity measurements, binding and dissociation were monitored using Biacore X (GE Healthcare UK Ltd.). The data were fitted using BIAevaluation software version 4.1 (GE Healthcare UK Ltd.) with the single-cycle kinetics support package (GE Healthcare UK Ltd.). Binding data were globally fit to the single-cycle kinetics 1:1 Langmuir binding model.

### Ligand-receptor binding assay.

An in vitro binding assay was performed to evaluate whether CBD-CNP binds to NPR-B. Equal amounts (10 pmol each for His-tag detection, and 90 pmol each for CBD-CNP) of CBD-CNP and His-tagged NPR-B (49 kDa) were incubated together at 4°C overnight. Then equilibrated Ni-NTA agarose beads (Qiagen) were mixed by inversion at 4°C for 1 hour to coprecipitate the protein complex. After 4 washes to remove nonspecific binding, the bound proteins were eluted with SDS sample buffer and heat-denatured at 98°C for 5 minutes. Proteins were separated by SDS-PAGE, transferred to a Poly Vinylidene Di-Fluoride (PVDF) membrane, and detected by Western blotting using anti-His-tag antibody (D291-3, MBL) and anti-CNP antibody (abx214951, Abbexa).

### Signal transduction assay with chondrosarcoma cells.

To evaluate whether CBD-CNP inhibits the FGF2-FGFR3 downstream signaling pathway via NPR-B, RCS cells, which were provided by Pavel Krejc (Medical Genetics Institute, Cedars-Sinai Medical Center), were cultured in Dulbecco’s Modified Eagle’s Medium (Nacalai Tesque) supplemented with 10% fetal bovine serum (Gibco) (23, 46). RCS cells were seeded at 1 × 10^5^ cells per well in a 6-well plate. The following day, the cells were maintained in serum-free medium. After 24 hours, the cells were treated with 10 ng/mL FGF2 (R&D systems) in the presence of 0.2 μM CNP-22 (Peptide Institute Inc.) or 0.2 μM CBD-CNP for 30 minutes. The cells were lysed in SDS sample buffer supplemented with proteinase inhibitors, heat-treated at 98°C for 5 minutes, and processed by SDS-PAGE then transferred to a PVDF membrane. The phosphorylation level of ERK1/2 was determined by Western blotting with anti-ERK1/2 antibody (#4695, CST), anti-phospho-ERK1/2 antibody (#4370, CST), and anti-histone H3 antibody (#9715, CST).

### Cell proliferation and Alcian blue staining.

RCS cells were seeded in a 6-well culture plate at a density of 1 × 10^5^ cells per well. The following day, the cells were treated with 10 ng/mL FGF2 in the presence of 0.2 μM CNP-22 or 0.2 μM CBD-CNP. After 72 hours, cell numbers were evaluated using the Cell Counting Kit-8 (Dojindo), and the cell proliferation rate was calculated by setting the number of cells with untreated control as 100%. For Alcian blue staining, the cells were fixed with methanol at –20°C for 30 minutes and stained overnight with 0.5% Alcian Blue 8 GX (Sigma) in 1 N HCl. The images were captured using a bright-field microscope (Olympus, APX100).

### Bone explant culture.

The tibiae of WT mice (FVB background, CLEA Japan) at embryonic day 16.5 were extracted under a microscope as described previously (37, 47), and cultured in a 24-well plate using BGJb medium (Invitrogen) supplemented with 0.2% bovine serum albumin and 150 μg/mL ascorbic acid. The tibiae were treated with 100 ng/mL FGF2 in the presence of 0.2 μM CNP-22 or 0.2 μM CBD-CNP for 4 days with daily medium changes. In a separate experiment to assess the prolonged effects of CBD-CNP in cartilage, only a single administration of 0.2 μM CNP-22 or 0.2 μM CBD-CNP was performed on the first day, whereas 100 ng/mL FGF2 was added daily. After 4 days, tibial lengths were measured and compared with contralateral untreated tibiae from the same patients. The longitudinal length of the tibia, defined as the length between the proximal and distal articular cartilages, was measured using ImageJ software (NIH).

### Spatial transcriptomics of bone explant cultures.

The tibiae from embryonic day 16.5 mice were cultured in a 24-well plate and divided into 2 groups (*n* = 3 per group): one treated with FGF2 alone and the other with FGF2 plus CBD-CNP group. In the FGF2 group, 100 ng/mL FGF2 was administered unilaterally. In the CBD-CNP group, one side received 100 ng/mL FGF2, whereas the opposite site was treated with 100 ng/mL FGF2 and 0.2 μM CBD-CNP for 4 days with daily medium changes. After treatment, tibiae were embedded in Tissue-Tek Optimal Cutting Temperature (OCT) compound (Sakura Finetek) on dry ice and stored at –80°C. OCT blocks were sectioned at 10 μm thickness, and RNA was extracted and assessed for quality using the RNeasy Mini Kit (Qiagen) and High Sensitivity RNA ScreenTape assay (Agilent Technologies), ensuring RNA integrity numbers > 7. Sections were mounted on 6.5 mm^2^ oligo-barcoded capture areas and tissue optimization was performed using the Visium Spatial Tissue Optimization Slide & Reagent Kit (10x Genomics) according to the manufacturer’s protocol, with 12 minutes identified as the optimal permeabilization time. After 10 μm tissue sections were fixed and stained with H&E, subsequent steps included tissue permeabilization, reverse transcription, second-strand cDNA synthesis, amplification, and library preparation, performed using the Visium Spatial Gene Expression Slide & Reagent Kit according to the manufacturer’s instructions. Libraries were profiled with the High Sensitivity D5000 ScreenTape assay (Agilent Technologies) and sequenced on a DNBSEQ-G400 (MGI), with a minimum of 50,000 read pairs per spot. Sequencing data were aligned to the mm10-2020-A reference genome using Space Ranger v.2.0.1 (10x Genomics). Gene set enrichment analysis was conducted to assess pathway and biological activity changes in the hypertrophic zone.

### In vivo fracture model.

A femoral fracture model was established in 9-week-old male C57BL/6J mice (Charles River Laboratories Japan Inc.), as previously described (21). In brief, a 4 mm left medial parapatellar incision was made, and a 0.5 mm hole was drilled into the intercondylar notch. A 0.2 mm tungsten guidewire was retrogradely inserted into the intramedullary canal, and a section of the femur was resected laterally. The guidewire was replaced with a 0.5 mm stainless steel screw to stabilize the fracture. Immediately after surgery, 5 mg of porcine dermal type I collagen powder (Nippi) containing 0.36 μmol/kg CNP-22, CBD-CNP, or PBS was injected at the fracture site (*n* = 8 per group). Four weeks after surgery, femurs were harvested and fixed in 4% paraformaldehyde at 4°C for 48 hours, followed by PBS storage. μCT imaging was performed using a microfocus x-ray CT system (inspeXio SMX-90CT; Shimadzu Co., Ltd.), with the following settings: accelerating voltage, 90 kV; current, 110 mA; voxel size, 20 μm/pixel; and matrix size, 1,024 × 1,024. A 10 mm region of interest (500 slices) was defined in the mid-femur. BV and BMC in the defined regions were quantified using a 3-dimensional image analysis software (Tri-3D-Bon, Ratoc System Engineering Co., Ltd.) as previously described (19–21). BMC of each femur was estimated by comparing the density measured in μCT images to a hydroxyapatite (HA) calibration curve, based on phantom images at 200, 300, 400, 500, 600, 700, and 800 mg HA/cm^3^. BV was defined using a threshold of ≥ 300 mg/cm^3^.

### Intraarticular injection of CBD-CNP in a mouse model of knee OA.

Knee OA was induced in a 10-week-old male FVB/NJcl mice (CLEA Japan) by medial meniscus ligamentotomy on the right hind limb, as previously described (48, 49). Two weeks post-surgery, intraarticular injections of 0.36 μmol/kg CNP-22, CBD-CNP, or PBS (10 μL each) were administered 3 times a week for 8 weeks. Three days after the final injection, hindlimbs were harvested for μCT and histological evaluation. The control data of μCT analysis were obtained from the contralateral side of the OA with PBS.

μCT scans were performed using the Skyscan 1176 (Bruker) as previously described (38, 39) with the following parameters: x-ray voltage, 50 kV; x-ray current, 500 μA; 0.5 mm Al filter; rotation step 0.5°; pixel size, 9 μm; and no frame averaging. Images were reconstructed using Skyscan NRecon software, and the images were analyzed using the 3-dimensional algorithms of the Skyscan CTAn software, according to the manufacturer’s instructions. The proximal medial tibial epiphysis was selected as the region of interest (ROI). To quantify the characteristics of the subchondral bone, the epiphysis was further separated into a cortical part (subchondral bone plate) and underlying a trabecular part. A subset (500 μm mediolateral width, 1,000 μm ventrodorsal length) of the weight-bearing region at the medial side of the tibial plateau was taken as the epiphyseal ROI according to the previous method (49). The trabecular thickness, number, separation, and BV/TV were calculated as indices of epiphyseal and metaphyseal trabecular compartments. The subchondral bone plate thickness was calculated using the same algorithm for trabecular thickness. BMD was assessed at the same sites as the BV/TV measurements, and the Skycan CT system was calibrated against 0.25 g/cm^3^ and 0.75 g/cm^3^ HA phantoms.

### Histological analysis.

Embryonic tibiae and adult murine knee joints were fixed in 4% paraformaldehyde at room temperature for 24 hours. Adult murine knee tissues were washed with PBS and decalcified in 10% ethylenediaminetetraacetic acid (EDTA) solution at 4°C for 3 weeks. The tissues were embedded in paraffin and sectioned at 3 μm for embryonic tibiae and 5 μm for adult knee joint tissues, then deparaffinized. The persistence of CNP and CBD-CNP in articular cartilage was assessed by DAB staining using ImmPRESS Horse Anti-Rabbit IgG Plus Polymer Kit (Vector Laboratories) and anti-CNP antibody (abx214951, Abbexa) at 1:100 dilution according to the manufacturer’s protocol. Rabbit anti-CBD antibody was prepared as previously reported (50) and used at 40 μg/mL. Stained sections were evaluated using a BZ-X710 Analyzer (Keyence).

### Statistics.

Data are expressed as mean ± SD. Comparisons between 2 groups were evaluated using the 2-tailed Student’s *t* test. Paired, 2-tailed *t* tests were used to compare tibial lengths within patients. For multiple comparisons of continuous measures between groups, 1-way ANOVA followed by Tukey’s post hoc test were applied to determine the statistical significance. Analyses were performed using SPSS version 26 software (IBM Corporation). *P* < 0.05 was considered statistically significant.

### Study approval.

All animal experimental procedures were approved by the Animal Care and Use Committee of Kitasato University (no. 2024-118) and Nagoya University (no. M250045-002) and were performed in compliance with the animal research guidelines.

### Data availability.

The values of all data in the graphs and all gene expression profiles are provided in the Supporting Data Values file. Visium spatial transcriptome sequencing data of fetal murine tibiae are deposited in the ArrayExpress database under accession code E-MTAB-16154.

## Author contributions

Designing research studies was contributed by KH, KU, MM, OM, and YH. Experiments were conducted by KH, KS, RE, YO, HS, and OM. Reagents were provided by RS, EA, TM, SK, and OM. Data were analyzed by KH, KS, RE, RS, YO, and HS. Manuscript was written by KH and KS. Manuscript was edited by YO, EA, MM, OM, and YH. KU, HT, SI, MM, OM, and YH supervised the research. Although KH and KS contributed equally to this study, we have designated KH as the first author because he is the inventor who conceived of CBD-CNP and contributed to the concept of this study.

## Funding support

- Japan Agency for Medical Research and Development (AMED; Tokyo, Japan) under the Translational Research Network Program (grant no. JP23ym0126810, JP24ym0126810) (to KH)
- Japan Society for the Promotion of Science (JSPS; Tokyo, Japan) Grant-in-Aid for Scientific Research (C) (grant no. JP23K06545) (to OM)
- JSPS Grant-in-Aid for Scientific Research (A) (grant no. JP24H00652) (to SK)

## Supplementary Material

Supplemental data

Unedited blot and gel images

Supporting data values

## Version 1 12/23/2025

In-Press Preview

## Version 2 02/09/2026

Electronic publication
