# Supplementary material for: Collagen-binding C-type natriuretic peptide enhances chondrogenesis and osteogenesis
Source: JCI Insight. 2025 Dec 23;11(3):e198959. doi: 10.1172/jci.insight.198959 (PMC12892892; doi:10.1172/jci.insight.198959)
Supplement: Supplemental data [file jciinsight-11-198959-s071.pdf]

gspgipGNEK LKEKENNDSS DKATVIPNFN TTMQGSLLGD DSRDYYSFEV KEEGEVNIEL
DKKDEFGVTW TLHPESNIND RITYGQVDGN KVSNKVKLRP GKYYLLVYKY SGSGNYELRV
NKGGSPGQEH PNARKYKGAN KKGLSKGCFG LKLDRIGSMS GLGC

■linker ■CBD ■spacer ■CNP-37

Supplemental Figure 1. Amino acid sequence of CBD-CNP.

Lowercase black text indicates a linker for production and purification of CBD-CNP. Red text indicates the CBD, yellow indicates the spacer, and green indicates the CNP-37 sequence.

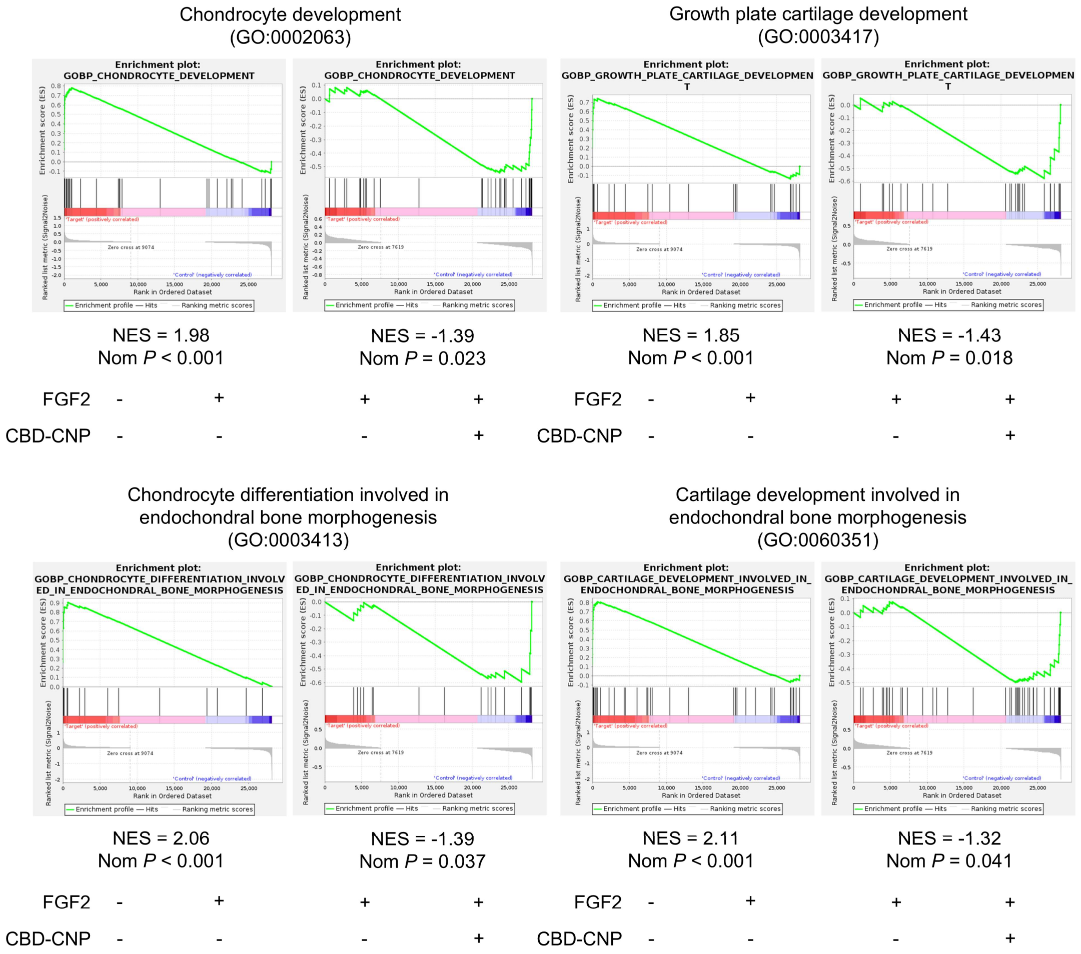

Supplemental Figure 2. Gene set enrichment analysis of hypertrophic zones in spatial transcriptomics.

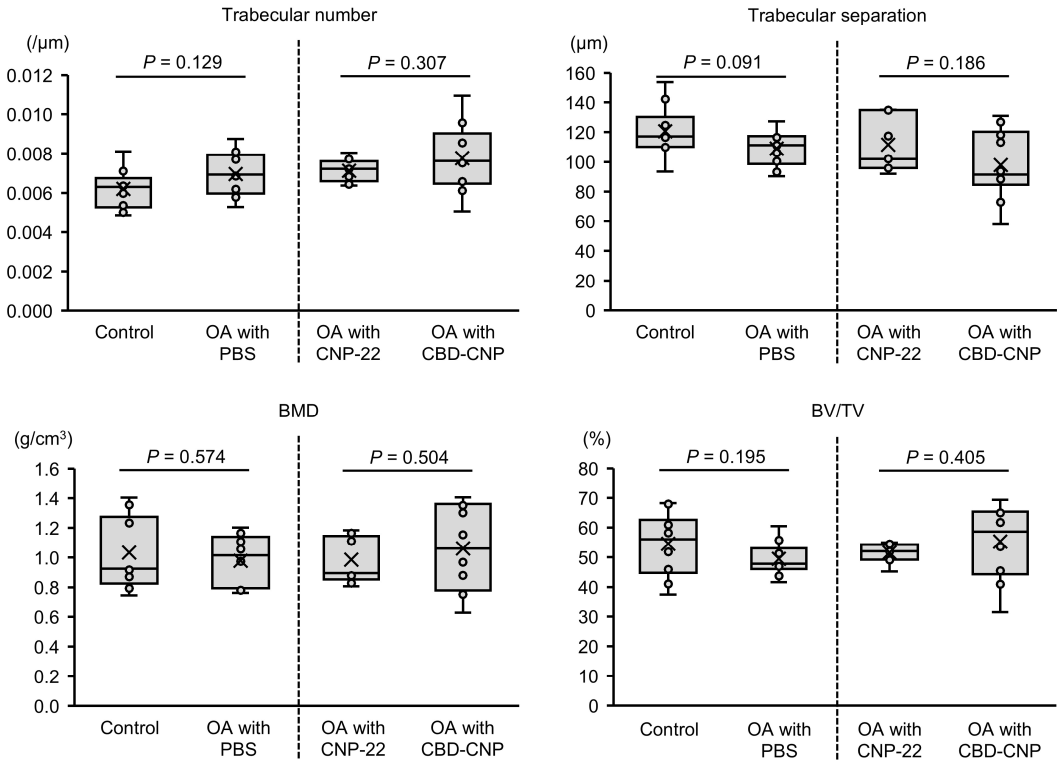

Supplemental Figure 3. Micro-CT analysis of intra-articular injection of CBD-CNP in a mouse model of knee OA.

Trabecular number, separation, BMD, and BV/TV were quantitatively analyzed using micro-CT images (n = 10 in control/OA with PBS, and OA with CBD-CNP groups, and n = 9 in OA with CNP-22 group). The data of control and OA with PBS were obtained from the same subjects. Statistical analysis was performed using the Student's *t*-test.

BMD, bone mineral density; BV/TV, bone volume/total volume.

Samples	a, b, c		d, e, f	
FGF2	(-)	(+)	(+)	(+)
CBD-CNP	(-)	(-)	(-)	(+)
Number of reads	392,366,240	387,854,984	385,730,642	397,337,908
Mean reads per spot	408,715	627,597	659,369	717,216
Median genes per spot	3,142	3,458	3,990	3,558
Median UMI counts per spot	12,509	14,367	16,906	14,540

UMI, unique molecular identifier.

Supplemental Table 1. Visium transcriptomics run data.
