# Supplementary figures and images for: Collagen-binding C-type natriuretic peptide enhances chondrogenesis and osteogenesis
Source: JCI Insight. 2025 Dec 23;11(3):e198959. doi: 10.1172/jci.insight.198959 (PMC12892892; doi:10.1172/jci.insight.198959)

Figure 2C

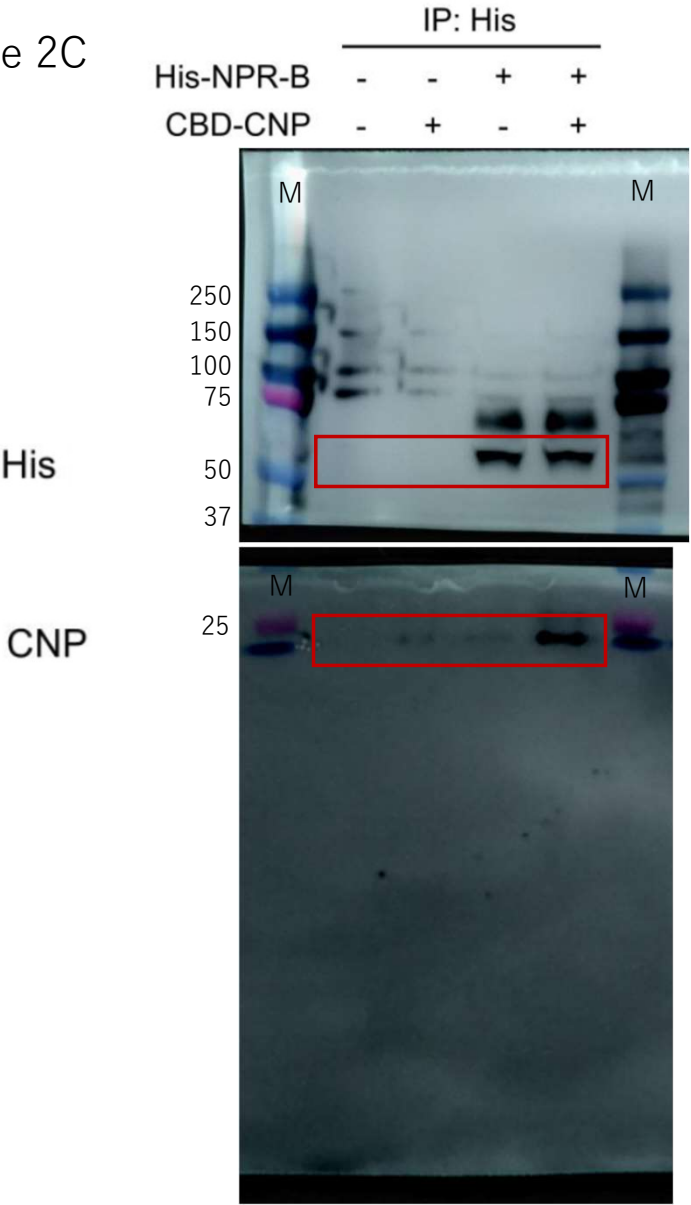

Figure 2D

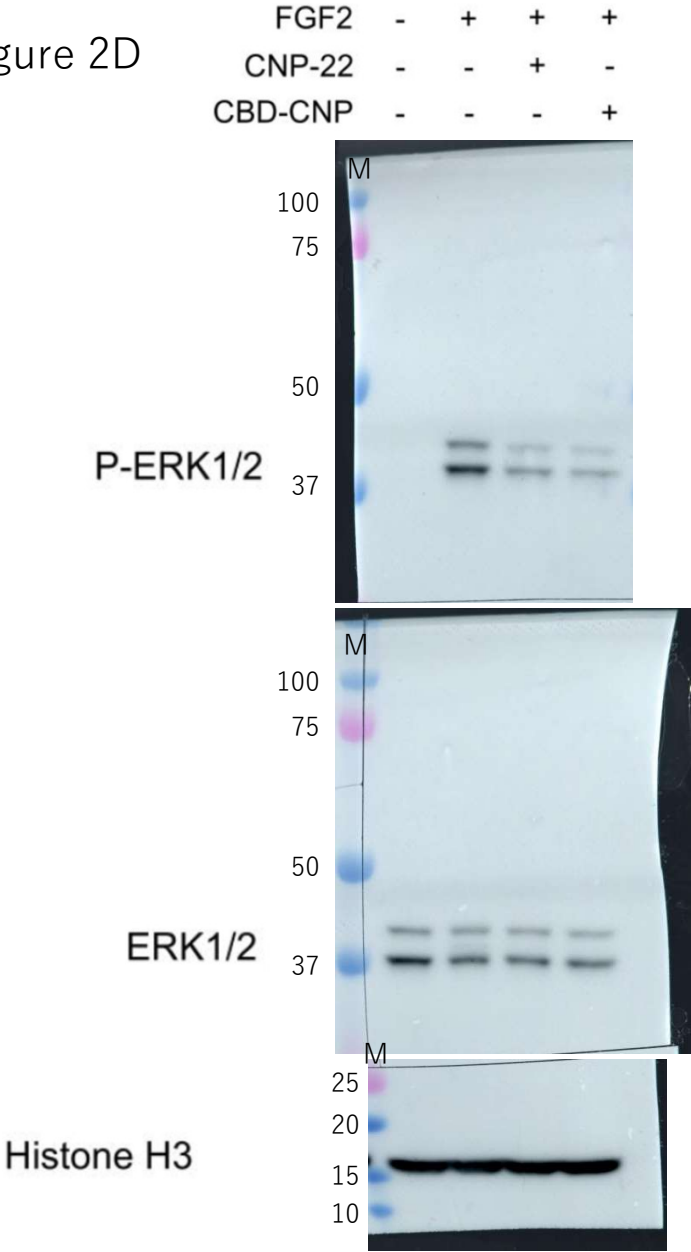

Supplement: Unedited blot and gel images [file jciinsight-11-198959-s072.pdf]
